# Repacking ‘Privacy’ for a Networked World

**DOI:** 10.1007/s10606-017-9276-y

**Published:** 2017-05-29

**Authors:** Andy Crabtree, Peter Tolmie, Will Knight

**Affiliations:** School of Computer Science, University of Nottingham, Jubilee Campus, Wollaton Road, Nottingham, NG8 1BB UK

**Keywords:** Privacy, Digital economy, Crisis in trust, Domestic digital privacy practices, Ethnomethodology

## Abstract

In this paper we examine the notion of privacy as promoted in the digital economy and how it has been taken up as a design challenge in the fields of CSCW, HCI and Ubiquitous Computing. Against these prevalent views we present an ethnomethodological study of digital privacy practices in 20 homes in the UK and France, concentrating in particular upon people’s use of passwords, their management of digital content, and the controls they exercise over the extent to which the online world at large can penetrate their everyday lives. In explicating digital privacy practices in the home we find an abiding methodological concern amongst members to manage the potential ‘attack surface’ *of* the digital *on* everyday life occasioned by interaction in and with the networked world. We also find, as a feature of this methodological preoccupation, that privacy dissolves into a heterogeneous array of relationship management practices. Accordingly we propose that ‘privacy’ has little utility as a focus for design, and suggest instead that a more productive way forward would be to concentrate on supporting people’s evident interest in managing their relationships in and with the networked world.

## Introduction

Digital applications and services increasingly rely on personal data. Indeed some authors go so far as to suggest that we are in the middle of a ‘personal data gold rush’ (Chaudry et al. 2015), with advertisers, Internet services, and governments alike all accumulating increasing amounts of personal data about ordinary citizens. It is thus suggested that personal data is the ‘new currency’ of the digital economy (Gates and Matthews 2014). That it is becoming so is not passing without comment or concern, however. A steady drip of media stories detailing the misuse and abuse of personal data is complemented by large-scale leaks, all of which combine to create what the World Economic Forum (WEF) describes as a global ‘crisis in trust’ (WEF 2014). The WEF suggests that key to remedying this crisis is rebalancing the ‘asymmetry of power’ that favours public and private institutions over individuals and thereby creates barriers to economic development. This, in turn, leads to an emphasis being placed on increased ‘privacy’ protection, as underscored by the introduction of new General Data Protection Regulation in Europe (EU 2016), a Utilization of Personal Data framework in Japan (Japan 2014), and a proposed Consumer Privacy Bill of Rights in the US (US 2012). Thus ‘privacy’ is seen as the key mechanism whereby the asymmetry of power inherent in the digital ecosystem can be rebalanced to foster trust in and ultimately engagement with the digital economy.

A concern with privacy is not new in CSCW and related areas of HCI and Ubiquitous Computing, where it is seen as a matter of primary concern driven by both the immediate and foreseeable impact of networked technologies, particularly ‘ubiquitous’ or ‘pervasive’ (mobile, location and sensing-based) technologies, on interaction. Running alongside this is a marked concern with the impact of social media on privacy, and challenges occasioned by increased online data harvesting (e.g., consumer analytics). Both of these technological threads lead to a pronounced interest in providing ‘users’ with enhanced control mechanisms to manage the flow of personal data in the digital economy and thereby protect their privacy. However, the empirical study presented here of digital privacy practices in the home suggests that within a domestic context at least people are more concerned with *relationship management* than they are with privacy per se. Thus we find a concern among the participants in our study with managing cohort-dependent risks, the accountability of action, and the manifold ‘intrusions’ into everyday life occasioned by their interactions in and with the networked world. These efforts at relationship management suggest that managing the potential ‘attack surface’ *of* the digital *on* everyday life is a primary concern to household members, and we explicate a range of fine-grained methods members have devised and employ to do this. Traditionally the notion of attack surfaces is associated with considerations of security, but in our studies participants did not distinguish strongly between matters falling under the rubric of ‘security’ and matters falling under the rubric of ‘privacy’ (such distinctions would appear to matter more to designers than members). Furthermore, on closer inspection, we find that for members the invocation of ‘privacy’ itself glosses a heterogeneous array of local practices for managing human relationships in and with the networked world. The upshot for design is that there is need to complement a concern with technological privacy mechanisms for controlling the flow of personal data with the development of relationship mechanisms that enable ‘users’ to manage their relationships in and with the networked world. This, we suggest, is key to building trust in and fostering engagement with the digital economy.

## Privacy in (some of) the design literature

The design literature on privacy is huge. A cursory search just of ‘proceedings’ in the ACM Digital Library returns 101 entries spanning 27 conferences, symposia, and workshops with ‘privacy’ in the title. If one looks to journals and other texts the number becomes significantly larger. It is hard to escape the impression that privacy is the most researched topic in computing, or contemporary computing at least. Summarising all of this literature would be a gargantuan task, so we limit our consideration here to CSCW and the associated fields of HCI and Ubiquitous Computing. Looking at the 69 conference proceedings listed for these in the ACM Digital Library (19 for CSCW, 36 for CHI and 14 for UbiComp), we find some 155 papers have ‘privacy’ in the title or abstract, not including adjunct proceedings, posters, panels, colloquium papers, and workshops. Thus we find some 29 papers on privacy in CSCW, 81 in CHI, and 45 in UbiComp. We also find some 32 special paper sessions or tracks on privacy in these conferences over the years, largely in CHI, covering a diverse array of privacy-related topics.

In order to take a closer look at how privacy is playing out in the literature we categorised each paper by *type* to identify whether or not a paper was 1) a *design* paper offering some kind of privacy framework for design or actual technological mechanism; or 2) provided an *evaluation* of a privacy mechanism; or 3) an *experiment* or empirical *study* of some kind. Of course many papers contain both a design and evaluation component, and we so restricted the evaluation category to those papers that concentrated on the evaluation component. We then examined the results to see what kinds of *trends* around privacy we might discern in the literature and found that the primary focus has been on *designing for privacy* and *studying privacy*, with design being particularly pronounced in UbiComp (51% of privacy papers in UbiComp fall into this category) and study being particularly pronounced in CSCW (59%) and CHI (49%). Taking a closer look, we categorised each paper by technological domain and found that top trending privacy domains in the literature we sampled were *ubiquitous computing* in general and in particular *location-based services* and *mobile devices*, followed by *social media*, and the *Internet*. There is pronounced interest in location-based services and mobile devices in UbiComp (respectively 33% and 18% of all UbiComp papers addressed privacy in these domains) and notably on design frameworks and mechanisms. Privacy interest in CHI is spread across domains (6% of all CHI papers addressed privacy and ubiquitous computing, 9% location-based services, 14% mobile devices, 14% social media, and 12% the Internet), though the predominant focus is on studies. CSCW predominantly focuses on social media (31% of all CSCW papers address privacy in this domain; 10% of papers focus on mobile devices and only 3% on location-based services), and especially on studying social media with respect to privacy. Thus, overall the top trending domains in which privacy has been explored in the literature we inspected are ubiquitous computing (9% of all papers) with particular respect to location-based services (15% of all papers) and mobile devices (14% of all papers), and social media (13% of all papers), with the Internet following up in fifth place (7% of all papers). Drilling down further, we examined each paper within the top trending domains to identify the specific privacy-related topic it addressed. We identified some 59 discrete topics, though found a pronounced interest in a) studying privacy and disclosure practices along with an increasing interest in how these issues play out within various relationships (e.g., friend, romantic, parental, teenager, etc.), and b) designing privacy settings, preference, profiles, policies, and personalisation mechanisms. We also found that the most pronounced topical interest in privacy by far lies in *location data*.

We found too that the vast majority of privacy-related papers in our sample (some 72%) offer *no* conception of privacy, but simply invoke privacy as a general ‘concern’ which either warrants some kind of investigation (a study or experiment) or the design and/or evaluation of technological mechanisms for managing privacy. Nonetheless, various understandings of privacy are to be found:

- *Privacy as control*. Perhaps the most common-sense understanding of privacy in the literature is that it is about the ability to *control* the flow of personal data. Often attributed to legal scholar Alan Westin (1967), this notion is articulated in various ways, including the extent to which individuals, groups, or institutions may control when, how, and to what extent information about them is: shared with others (Tang et al. 2011), known to others (Wang et al. 2016), or communicated to others (Page et al. 2013). The control thesis may also be applied to how information and its visibility is limited (Shoemaker and Inkpen 2001, Williamson et al. 2010), filtered (Smith et al. 2012), directed (Gorm and Shklovski 2016), or used (Karat et al. 2006). On this view then privacy amounts to an individual or organisational claim, right, and ability to limit, filter, and control the flow and use of personal data.
- *Privacy as boundary management*. Based upon the theoretical work of social psychologist Irwin Altman (1975), privacy is here construed of as: a dynamic boundary regulation process where selective access to the self is governed by individuals-in-interaction according to circumstance (Palen and Dourish 2003); a process of continuous negotiation (Iachello and Abowd 2005); the intersubjective management of boundaries that are continually in tension with each other (Khalil and Connelly 2006); dynamically optimising the level of openness and closeness of self to others (Oulasvirta et al. 2012); or regulating physical, informational or social access (Page et al. 2013). Recent work (Kumar and Schoenebeck 2015, Cho and Filippova 2016, Blackwell et al. 2016, Jia and Xu 2016) has also seen the introduction of Sandra Petrioni’s (2002) Communication Privacy Management (CPM) theory, which attempts to extend Altman’s work by specifying how boundaries are regulated through rule development. On this view, then, privacy is an interactive process of interpersonal boundary management governed by rules controlling data access and disclosure.
- *Privacy as contextual integrity*. Like CPM, this is a more recent addition to understandings of privacy in CSCW and related areas. Drawing on media, culture and communications theorist Helen Nissenbaum’s (2004) concept of contextual integrity, privacy is here viewed as a collective-level property governed by context-specific norms of information flow (Cho and Filippova 2016). These norms demand that information gathering and dissemination be appropriate to specific contexts and obey the governing norms of distribution operating within them (Shi et al. 2013), with privacy being the optimisation of negotiating these (Guha and Wicker 2015). Some argue that there is no aspect of human life that is not governed by context-specific norms of information flow (Barkhuus 2012), which shape situated practices of disclosure (Papoutsi and Ian Brown 2015). Here privacy amounts to shared context specific social norms or values, controlling what kind of information it is appropriate to collect and disclose and shaping privacy practices in particular settings.
- *Privacy as paradox, trade-off, and concern.* Some researchers have identified discrepancies between people’s stated privacy attitudes and what they actually disclose (Kostakos et al. 2011), a phenomenon often referred to as the ‘privacy paradox’. Phelan et al. (2016) account for the paradox in terms of a divergence between the ‘privacy calculus’ people employ in practice and reported behaviour. This view positions people as rational entities who see privacy as a commodity and weigh disclosure decisions in terms of a risk-benefit trade-off (e.g., Knijnenburg et al. 2013, Wisniewski et al. 2015). This, in turn, drives an interest in ‘privacy concern’, that is the measurement of factors impacting the disclosure of personal data, including factors implicated in data collection, errors in and improper access to personal data, and unauthorised secondary use (Smith et al. 1996). While Phelan et al. (2016) argue that it is unrealistic to expect that people will behave rationally when making privacy-related decisions, this conceptualisation of privacy nevertheless motivates and drives a significant body of design research.
- *Privacy as a protective measure*. Given the apparent divergence of views some argue that privacy is an indefinable (Langheinrich 2001) and indeed personal notion (Jiang et al. 2002). Bellotti and Sellen suggested as far back as 1993, “it would be useful to have a practical working definition of the concept but unfortunately it is difficult to define this notion in more than an intuitive fashion”. This, however, does not set aside allied concerns with the potential ‘threat’ of networked technologies to individual freedom and independence (Vasalou et al. 2012), and the turn to privacy-seeking behaviours (Oulasvirta et al. 2012). The threat-model leads to a view of privacy as a *protective measure*. Thus designers have proposed privacy-by-design frameworks and approaches (e.g., Langheinrich 2001, Scipioni 2012), particularly in the field of ubiquitous computing. Some have even drawn up taxonomies of information harm (Solove 2008). This conception of privacy does not seek to define an everyday phenomenon, but like the privacy paradox, motivates and drives a substantial portion of design research.

Our cursory review of the privacy literature in CSCW and related areas reveals several discrete trends. That the predominant focus is on *designing for privacy* and *studying privacy*. That the top trending privacy domains in which privacy is explored are *ubiquitous computing*, and particularly *location-based services* and *mobile devices*, followed by *social media*, and the *Internet*. That CSCW predominantly focuses on studying topics related to social media, but the predominant interest in the broader ACM-related literature is with *location-based data*. That most privacy-related papers are motivated by ‘concern’ in general, rather than by particular understandings of privacy. That particular understandings of privacy are varied but focus, on the one hand, on various aspects of *privacy as control* (of information flow and use, boundary management, and context-specific norms ordering the flow of data), and on the other as something that cannot be defined but nevertheless warrants design research given the apparent *paradox* between people’s attitudes and behaviours and the potential *threat* of networked technology to individual autonomy.^1^

## Studying privacy in everyday life

In a bid to try an understand what privacy looks like from the perspective of everyday life we undertook a study of domestic digital privacy practices in 20 households in the UK and France, leveraging the social networks we are members of to find participants willing to engage in our study. Notably, not everyone we asked was willing to participate, feeling the study would be inappropriate given their personal relationship to ourselves. Engaging with us in a professional capacity thus made some people uncomfortable and unwilling to engage in the research, and it might be said that privacy became a barrier to research as others have found before us (Tang et al. 2006). Nevertheless, and with little effort, we did find people who were willing to engage in the research, and indeed more than we could handle, including people who were complete strangers to us but came to engage via friends and colleagues. Demographically our participants came from a wide array of occupational backgrounds and household situations, as can be seen in Table 1 (the occupational descriptions and ages of participants is based on their own accounts and participants in the UK and France are denoted UK and FR respectively). We took the opportunity of studying people in France for the added diversity it might bring to our sample. However, despite the obvious language differences, the most notable thing about the French cohort was the extent to which its members exhibited exactly the same methodological preoccupations with regard to managing their digital privacy as the UK participants.

**Table 1:** Participants in the study of digital privacy practices.

Participants	
Household 1	Husband (60s) and wife (60s), living with youngest son (30s). Husband runs his own export business from home. Wife is a reflexologist who also runs her practice from home. (UK)
Household 2	Couple, male (40s) and female (30s), living with a 6 year old daughter. Male is a theatrical performer and producer. Female is a part-time classroom assistant. (FR)
Household 3	Husband (50s) and wife (40s), living with a 6 year old daughter and baby boy. Husband is an academic working in a university computer science department. Wife is a secondary school teacher. (UK)
Household 4	Female (50s), living with teenage daughter and who has 2 older sons at university. Healthcare professional. (FR)
Household 5	Female (22), living at home with her parents and two siblings. Student studying for a fine arts degree. (FR)
Household 6	Couple, male (20s) and female (20s), expecting first child. Male is an IT support engineer. Female is an office worker. (UK)
Household 7	Female (40s), living with son and daughter. High school philosophy teacher. (FR)
Household 8	Couple, male (24), female (22). Male is an insurance call centre worker. Female is a full time masters’ student. (UK)
Household 9	Female (30s), living with male partner and 2 young children. Works in fashion. (UK)
Household 10	Husband (30s) and wife (30s), living with a 2 year old daughter and a baby. Husband is social science researcher. Wife works for local council. (UK)
Household 11	Female (16), living with her mother and brother. College student. (FR)
Household 12	Couple, male (30s), female (30s). Male is full time student, studying ergonomics. Female is a full time student, studying psychology. (UK)
Household 13	Male (40s), living with wife (40s) and 2 children (girl 17 and boy 21). Software developer. (UK)
Household 14	Female (13), living with parents and 2 siblings. Schoolgirl. (FR)
Household 15	Husband (50s) and wife (50s), living with 3 children (one teenage girl, one female in early 20s and male in his early 20s), also have another son who lives elsewhere. Male is a consultant. Female is a housewife and artist. (FR)
Household 16	Male (30s), living with his parents. Works as a freelance dance music curator for YouTube, Google and MTV. (UK)
Household 17	Couple, male (50s), female (50s), have 3 children but none live with them. Male works in healthcare dealing with patient safety. Female is a radiographer’s assistant. (UK)
Household 18	Husband (30s) and wife (30s), living with 2 daughters aged 6 and 9. Husband works as researcher and software developer in a university. Wife is primary school support worker. (UK)
Household 19	Male (23), living alone. Student doing business studies and modern languages. (FR)
Household 20	Husband (70s), lives with his wife (60s). Retired. (UK)

This gave us an eclectic sample with which to explore how privacy is socially constituted in everyday life from the perspectives of a range of people of different ages, different walks of life, and different circumstances, who have different occupations and interests, and live in different environments that span town, city and countryside in two different countries.

Everyday life is of course replete with practices whereby people manage what gets to be seen, touched, handled, or used by other people. Artifices abound for accomplishing this from open and shut doors, to raised or lowered voices, to proximity or distance, to using certain places or going to certain locations, to actively employing locks and keys and physical barriers. Touching or handling objects may have a set of clearly articulated rights of access, or the rights may be tacit and known-in-common through the membership of particular cohorts of people such as the inhabitants of a home or co-workers or fellow travellers. And certain things may be spoken of to some but not to others, and in a variety of ways. In undertaking our study we wanted to understand how such practices map into the digital sphere. Before we could begin our exploration we were required to gain ethics clearance. In making our case for the study we agreed that no digital data would be gathered – i.e., that we would not collect content from participants’ devices (browsing history, communications, etc.) but would instead focus on eliciting members’ accounts of their digital privacy practices. As we did not seek to constrain what our participants might show and tell us, we did not employ a structured methodology. Instead we loosely formulated a handful of broad questions that on the face of it speak to digital privacy and employed these to open up and drive a free-flowing conversation with our participants. Thus, and for example, we asked our participants about the use of passwords with respect to their devices, home network, applications, and digital content. We wanted to know who and what they were granting access to, on what grounds, and the circumstances in which access might be granted. In short we wanted to know what managing privacy looks like as a practical matter and what people’s ordinary everyday digital privacy practices consist of.

Our approach therefore involved ‘interviewing’ people or, to be more accurate sitting down with them in their homes, drinking tea, eating biscuits and the like, and casually discussing what privacy might be about as a practical matter in their mundane dealings with the networked world. We spoke to whomever in the home was willing to engage with us, after all persons cannot compelled to speak just because you are doing research. Thus in some households we interviewed whole family groups or couples, in others only a single inhabitant wanted to speak to with us. Where possible couples and family members were interviewed together in order to understand how they accounted for privacy to one another as well as to us. Engaging participants in their homes meant that wherever it was practicable the devices being talked about were also to-hand, and could be drawn on to prompt detailed discussion and to demonstrate specific practices. Some of the ‘interviews’ were as brief as 40 min; others lasted for over an hour and a half. But in all cases we had the same end in mind: to elaborate in concrete detail people’s mundane digital privacy practices. Our conversations with participants were recorded on audio and subsequently transcribed, and the transcripts were analysed to identify the practical sociological and thus ‘collaborative’ nature of digital privacy practice in everyday life.

It may, on the surface, appear to some that our approach lacks rigor and the question be begged, how can talking to people in an informal and unstructured way provide for sound analytic results? However, we take seriously Harold Garfinkel’s (1967) pronouncement that,

“There is a feature of members’ accounts that for them is of such singular and prevailing relevance that it controls other features in their specific character as recognisable, rational features of practical sociological inquiries. The feature is this. With respect to the problematic character of their inquiries, members take for granted that a member must ‘know’ the settings in which he is to operate if his practices are to serve as measures to bring particular, located features of these settings to recognisable account. *They treat as the most passing matter of fact that members’ accounts, of every sort, in all their logical modes, with all of their uses, and for every method of their assembly are constituent features of the settings they make observable.*” (our emphasis)

In other words, in accounting for their privacy practices, members were not merely telling disinterested stories delivered in the manner of an abstract narrator. Instead the very act of describing and demonstrating their digital privacy practices was constituted as a part of those practices as well. Thus in telling us about privacy management they were displaying the local ways in which privacy management was done in their domestic lives too. This is what Pollner (2012) describes as the ‘endogenous reflexivity’ of members’ accounts and it is the practices or ‘methods’ reflexively manifest in the telling that we explicate below.

### Some methodological issues

Before we move on to present the results of our study we first need to examine some issues that have arisen during discussion of this paper. One concern raised related to our participants, who might be viewed as providing a limited ‘sample’ rather than an eclectic mix given the absence of same-gender, religious, or wealthy families, the working-class, etc. Now one might wonder why there is need to appeal to class, gender, religion, wealth, etc., rather than the mix of categories our participants used to describe themselves. What’s wrong with reflexologist, theatrical producer, classroom assistant, housewife, dance music curator, schoolgirl, etc.? The answer from ethnomethodology’s point of view lies in the conventional logic of social science inquiry, which seeks to privilege certain categories as key to understanding social order. Thus, class, gender, religion, wealth, etc., purportedly allow us to view the operation of general cultural matters in and across diverse settings: distributions of women in work, or various sectors of work, correlations between ethnicity and wealth, etc. (see Button et al. 2015 for further elaboration). This contrasts sharply with ethnomethodology’s reasoning about sampling and generalisation (Sacks 1992), a point we have previously elaborated at length in CSCW demonstrating that generic phenomenon of order may be identified from very small samples indeed (Crabtree et al. 2013, Crabtree and Tolmie 2016). What enables this is the elaboration of members’ methods, which, following Sacks et al. (1974), may be generally construed of as ‘context and cohort independent’. That is to say that it matters no more to understanding the methods that members use to order digital privacy that our participants are reflexologists, artists, housewives or school children of this or that class, gender, religion or income bracket, than such categorisations matter to understanding the orderliness of the highway, how people drive together, cross busy roads, navigate the streets, queue to buy coffee and doughnuts or do any of the manifold things that make up everyday life. The point to appreciate is that it is *not the actor that matters* (Sharrock and Button 1991), it is the action and the methods that order it.

Another concern was that our approach might be seen to lack a clearly planned methodology, rendering our observations harder to interpret, evaluate or understand. In particular, a question was posed as to whether we might need to be more reflexive about our investigatory practices, clarifying whether or not the same topics were raised in every household, what more was discussed, and that we elaborate for the reader just what was asked, when, and how. In answer to this we would emphasize that we didn’t raise the same topics in each household, as we weren’t administering a compliance document (e.g., a pre-scripted set of questions). Rather than pre-figure the matter we observed “Sacks’ gloss” (Garfinkel and Wieder 1992), which is to say that we wanted our participants to tell us what we could be talking about when we talk about privacy. We wanted them to make the phenomenon accountable, and accountable in very local, setting specific ways, as it is encountered by them in their everyday lives. So our questions were mere primers, picked up or discounted as our participants saw fit, and opened up on the fly by them and us with respect to their everyday actions and interactions in and with the networked world. In detailing the accounts participants offered, and there are a great many of them in this paper, it is plain to see *what* was discussed along with many of the particular questions that were posed too. Was there *more* to our discussions than is documented here? Of course, a discussion is not a paper, and both do different jobs of work, e.g., one elaborates a topic of interest, the other conveys key aspects of what was found. *When* did our conversations take place? In the morning, afternoon or evening as participants preferred. What about *how* we engaged them in conversation? If we were to take that question seriously then we might find ourselves detailing our conversational practices and it would be found that they consist of very mundane practices of the kind elaborated by Conversation Analysis (Sacks et al., 1974). Would it, however, really help the reader interpret, evaluate or understand our observations to unpack the ‘indexical’ (Garfinkel 1967) character of our talk and provide a ‘referentially’ (Pollner 1991) reflexive account of how our turns at talk were distributed, topics of talk were broached, and conversations steered by either party? To borrow from Lynch (2000),

“Studies of ‘our own’ investigative practices may, in some cases, be interesting, insightful and cleverly written, or they may come across as tedious, pretentious and unrevealing … reflexivity in general offers no guarantee of insight or revelation.”

In our view, then, greater reflexivity in this case would not be productive. Instead we would like to note that the veracity of our study – what enables others to interpret, evaluate, or understand our observations - does not turn upon academic reflection on our investigative practices but rather on the endogenous reflexivity *of members’ accounts*, for it is these accounts that make the phenomenon to hand observable.

A further worry was that our account of our analytic method might appear undisciplined and leave important analytic details unsaid, particularly regarding textual analysis. However, we are not engaged in textual analysis: we are analysing members’ accounts. Recognising this is the first step in our analysis, a point underscored by Garfinkel above. The second step, and again following Garfinkel, is to recognise that members’ accounts are constituent features of the settings they make observable, which means that in telling us about privacy management our participants were displaying how privacy management is done in their domestic lives too. The third step is to work through participants’ accounts to find out what is accountably involved in doing privacy management, which involves explicating and describing the practices or ‘methods’ elaborated by our participants in their accounts. So, in treating transcripts as accounts, and accounts as constituent features of the settings they make observable, elaborating in the telling how our participants do privacy management, those accounts were examined for their innate methodological properties (Benson and Hughes 1991), the results of which are conveyed below. One more methodological issue must be touched on before we arrive at those results, which is that the data we present below might appear to suffer from selection bias. Our participants provided us with a wealth of material, far more than we can treat here, and so we focus quite explicitly on the most ‘grossly observable’ features of privacy as manifest in our participants local, situated accounts – i.e., that they exploit an array of methods to manage the potential attack surface of the digital on everyday life. It is towards explicating this methodological phenomenon and making it instructably observable that this paper is specifically oriented.

## Privacy in practice

One of the most notable features of participants situated accounts of domestic digital privacy management, and a matter we shall return to in due course, is that privacy as a topic breaks down into a wide variety of different and essentially unrelated local practices once you start to examine what people actually *do*. Nonetheless, what we want to do in the following materials is articulate an abiding concern that members displayed in heterogeneous ways to manage the potential attack surface of the digital and how this plays out in their everyday lives.

### Passwords and privacy

The first thing we note about domestic digital privacy practices is that only one of our participants exhibited the slightest *concern* about location-based data, and could see ‘no reason’ for their phone to track ‘where I’m going in everyday life’. This is not to say that our participants did not use location-based services, many did of them did, only that location-based services were not seen or treated as problematic. Thus our participants simply turned them off if they didn’t want to use them, and many did so to prolong battery life. Location data was not discussed as something that needed any particular management with regard to what was shared with other people. Indeed, the only time that location was mentioned in regard to privacy was with respect to passwords, and rather surprisingly so:

Paul: I’m not particularly fussed about setting up passwords and things. I mean there’s no threat of network hijacking here. We live in the middle of the countryside, miles away from another house, it’s just not an issue.

As Kaye (2011) points out passwords are, from the perspective of systems design at least, seen as a key privacy mechanism yet it is not at all uncommon for passwords to be shared. Indeed, in our own study we also found that the use of passwords was routinely *suspended* under the practical auspices of ‘convenience’. As one participant put it with respect to his phone, for example,

I know it’s got my life on it but I look at it about thirty times a day or something and if I’m having do that (mimes punching in PIN number), you know!

This was not the only example of password suspension in our study, particularly with regard to devices that stay within the home such as desktop PCs, tablets and media servers. That users do not religiously employ passwords, and apparently share them with abandon, is a constant source of complaint for security experts. Nonetheless, they are *not* religiously employed, and *are* often shared, so we sought to understand on what grounds they are used and/or shared, or not as the case may be, across their networks, devices, accounts, and content.

Unsurprisingly it turns out that the use of passwords is occasioned by a wide variety of practical issues and concerns. Thus some participants told us they used them because their devices ‘asked them’ for one. Often they had ‘no choice’ because they were using devices supplied by the organisations they worked for or devices simply could not be used without them. Others reported using passwords for personalisation, thus logging into a Hotmail or Google account “gets you everything”. Loss or theft was an often-cited reason for using passwords on mobile devices. Here participants felt that passwords might not only protect their data from uncontrolled access but also provide a motive for returning a mobile device to its owner. Managing access to devices and networks was another reason given for using passwords in general. However, passwords were not generally used because it was thought to be ‘good practice’. Only one participant, an IT support engineer, accounted for password use on this basis. For the main part we found that the use of passwords was *contingently occasioned* by the *potential risks* that attach to *particular cohorts*. As one couple put it,

Mike: The PC upstairs occasionally has a password. It usually doesn’t. It’s in the back room. The last time I put a code on was when we had a decorator in to do that room. I’ve done it once or twice when we’ve had guests staying …

Alice: Yeah, when my nephew comes, ‘cause he just logs into everything …

Fieldworker: It kind of depends on the guest?

Mike: Yeah.

Fieldworker: ‘cause you see it as a potential risk?

Mike: Yeah.

Fieldworker: What would be a potential risk?

Mike: Basically, er, adult material on there. So potential embarrassment I guess. With the decorator guy, it was more the general principle. There’s personal information on there …

Alice: Bank details and stuff, so we don’t want them …

Mike: Yeah. Whereas, if it was like family staying there, it’s more like the scenario where they just use the PC for something and stumble across a folder I’d rather they don’t stumble across.

Passwords are, then, seen and treated not so much as a blanket privacy mechanism but rather as a means of managing specific risks: e.g., the risk of exposing a child to inappropriate content, the risk of causing embarrassment to guest and self alike, the risk of exposing personal and even sensitive data to those who have no business looking at it, etc. This use of passwords to contingently manage foreseeable cohort-dependent risks ran throughout our data, particularly with regard to children.

Kit: On the television we’ve got one. You know, like on Netflix. Because when the children - ‘cause Mary’s sort of nine, she can go and choose what she wants to watch - but there’s certain things, if it’s above a PG, you need a PIN.

Tim: We only did that when she had friends round.

Kit: Yeah, that’s when she had a sleep over.

Tim: Before that, didn’t bother.

Kit: She had some friends over for a sleep over and I thought, oh – ‘cause we know that Mary wouldn’t go on to other things - but we thought, not sure what friends would do. We thought we’d just put that on. Just left it there now. So that’s passworded.

The risk of exposing children to inappropriate content is writ large in participant’s accounts and drives the use of passwords across all manner of networked devices and the concomitant use of managed accounts that restrict access to online content. In this respect then passwords are invoked with respect to parental responsibility and employed methodically as ‘gateway’ devices by parents or guardians, just as they are employed methodically to manage potential risks occasioned by guests or others more removed from the household cohort.

The methodical use of passwords as gateway devices enabling risk management is particularly pronounced when it comes to sharing, which is again contingently occasioned by a broad range of cohort-dependencies. Thus we find that our participants employed passwords alongside a range of *risk management strategies* within the various accountable relationships they had with ‘children’, ‘partners’, ‘family’, ‘friends’, ‘friends of the kids’, ‘guests’, ‘tradesmen’, ‘clients’, etc. First and foremost amongst these risk management strategies was ‘the front door’. This is not to say that being allowed through the front door warrants password sharing, but that it is an important premise or criteria for making such judgements: if you are not allowed to enter the home the gate is generally barred (though we are aware that network access may occasionally and with good reason be shared with neighbours, see Crabtree et al. 2012). Getting through the gate also turns upon the accountable *relationship* someone has to the members of the home. Thus, we found that ‘family’, ‘friends’, ‘friends of the kids’ (specifically teenage kids) and ‘baby-sitters’ were routinely given passwords to access networks, devices, and applications whereas ‘tradesmen’ and ‘clients’ of home-workers were not, and that this accountably turned upon ‘trust’.

David: The home you can police through other means – non-digital policing - so the doorway, if you’re in the house you have some kind of trust. If you’re like a tradesman then we might still, like put the password on the PC upstairs, but otherwise everyone is people we know and have some kind of trust with.

This is not to say that various categories of ‘visitor’ were untrustworthy, but that allowing them through the gate was occasioned by different orders of their accountability to relationships of ‘trust’. In cases where visiting was premised upon purely professional criteria we thus found that gateway access was more heavily controlled (with participants entering passwords into their own devices to enable application use) and even monitored (with participants ‘sticking around’ to ensure visitors didn’t do anything they didn’t want them to do).

The front door and ‘trust’ may be sufficient to manage network access with regards to ‘family’, ‘friends’ and ‘guests’, but that does not mean that the members of these cohorts have blanket rights of access. Rather, we find that different gateways are in operation and that access to devices, applications, and content is predicated on *cohort relevance*. Thus we find, for example, that partners routinely access one another’s personal devices because doing so is relevant to the relationships they have with one another. We find that ‘families’ routinely share passwords to enable members of the cohort to access applications and content, and that this applies to both static and mobile devices, for a wide variety of reasons including entertainment, way-finding, cost, and ease of communication. And we find that ‘household members’ generally share passwords, even passwords to sensitive data, to collaboratively handle a contingent array of domestic matters.

Sam: Liam knows some of my bank stuff, because I have to get him to buy things from time to time. He knows the PIN code for several of my bank accounts.

Fieldworker: So he’s of an age where you trust him with that?

Sam: Yeah, well because he’s got his own bank account and is competent in using it I figure he’s going to understand how to use mine.

Whether paying for goods and services for others, or posting items online for others, or sending emails on behalf of others, etc., the demands of domestic life routinely occasion password sharing. It is not done blindly, however, but on a cohort-relevant basis, which further enables *selective* gateway access and the concomitant management of risk.

That people share passwords with one another, particularly for devices, applications and content, does not mean that anything goes.

Joe: My wife might use my phone if it’s handy, or I might use hers, you know. It’s not a big deal for us. But my daughter [who is 17] has got a PIN number on hers, and I think my son [who is 21] has as well. He’s got his locked.

Fieldworker: You don’t know the PINs?

Joe: No, no. They have all their feeds coming in, Snapchat and Twitter and god knows what.

Fieldworker: Liam and Erin [late-teens and early twenties respectively], you wouldn’t know their passwords?

Carrie: No. We consider their stuff as private. We don’t need to nose in.

As Joe and Carrie make perspicuous, people employ *cohort-relevant access controls* that may be driven by a prima facie concern with ‘privacy’, as in the above example where one’s children are concerned, but are governed more generally by accountable expectations of *appropriate relationship-relevant* behaviour. Thus we find that while partners may routinely access one another’s personal devices, they do not necessarily know their children’s passwords (which very much depends on their age and the expectations that go along with that), and neither do they necessarily share passwords for various application accounts. It is not that they are being ‘private’ - as partner after partner told us they have ‘nothing to hide’ – it is that what is done is *not relevant*, and is seen as *not relevant* by both parties, and that accessing it would therefore be inappropriate.

Gene: You know I haven’t got anything to hide, and I don’t think my wife does so we’re kind of fairly open. I wouldn’t mind if she read my messages, you know, we’re not hugely secretive. We try to be open with each other.

Fieldworker: But you’re not actually trawling through one another’s mail either?

Gene: No. There’s an etiquette I suppose, and most email’s pretty dull anyway isn’t it. I wouldn’t look at my wife’s email and social media.

Cohort-relevance underpins sharing passwords, and not sharing them. Whether ‘partners’ or ‘parents and children’ or various categories of ‘other’ entering the home, cohort-relevance is determined by a host of accountable expectations regarding relationship-relevant behaviour. These expectations shape selective gateway access to networks, devices, applications and content and thus enable people to manage a contingent array of cohort-dependent risks that accompany interaction in a networked world. It might thus be said that in devising fine-grained methods of gateway control our participants minimise the potential ‘attack surface’ *of* the digital *on* their everyday lives and thus manage the potentially malicious or unintended consequences of interaction in *their* networked world. It is notable that these methods are not wholly devised to manage ‘attacks’ on their privacy. Indeed, ‘privacy’ was only occasionally invoked to account for the use of these methods, and even then it frequently glossed a range of alternative concerns: child safety, good parenting, avoiding embarrassment, doing things for others, behaving appropriately, being a good host, etc. It would thus appear that gateway management is wrapped up in a locally contingent array of mundane concerns involved in the conduct of *interpersonal relationships*, and that it is attack on the accountable conduct of these relationships that our participants seek to minimise if not prevent entirely. This methodological concern with relationship management is also evident in our participants’ management of digital content.

### Digital content and privacy

Most of the households we spoke to stored a wide range of personal content. This included records of passwords stored in various format: some used a personal code or mnemonic, and a few kept digital records that were encrypted, but most used physical formats (hand written notes) and stored them in a variety of personal locations that are typically hard for outsiders to access. Content also included financial records of all kinds, an array of ‘important’ documents (insurance certificates, scans of passports, national insurance numbers, television licenses, receipts, work-in-progress, etc.), family videos and photographs, and occasionally for a few, activity data generated by smartphones and wearable devices. These data were distributed across various devices and servers. The privacy of financial records, particularly bank details, was of common concern across our participants and these were typically stored locally, rather than online. However, a great many important documents were stored ‘out there’ on email servers, as this is the mechanism whereby many such documents are delivered, and on online solutions (OneDrive, Google Drive and Dropbox were frequently mentioned).

Joe: It’s a whole archive of my photographs and stuff that I’m entrusting to Microsoft.

Fieldworker: Trust is the key word there.

Joe: Yeah, it’s just trust. Purely that really. But what do you do with it though? Do you download a copy onto a hard drive and stick it in a safe somewhere in your house, you know? And how do you manage then to keep updating that?

Joe: I suppose if something horrible did happen, like Microsoft wiped all my data, how would that affect my life? Ultimately it probably wouldn’t really.

Fieldworker: So you reason about the risk?

Joe: Sometimes. I know there’s risk there and I know I’m placing a lot of trust in these big companies, but then who do you place your trust in? If the government, like the inland revenue, said, oh we’ve got a secure vault now, we can store your data, would you trust them more than you trust Microsoft? They could pass your information on to the security services.

Fieldworker: Sounds like you’d trust them less?

Joe: I think I would trust the government less to secure my data. Companies like Microsoft and Google have got their reputation, haven’t they, and that’s what their income’s based on. So if they break trust with millions of people around the world then that’s really going to affect them. So I suppose there’s that incentive for them.

Joe’s account, which is by no means unusual, makes it perspicuous in the first instance that the use of online solutions provides a practical way of *managing* collections of personal data. It is visible too that in using online solutions, people are not unaware of potential risks in putting personal data out there, but that these are mitigated by ‘trust’. Furthermore we can see that ‘trust’ is *not* groundless, but predicated on providers having a reputational and financial incentive to keep personal data secure. And we can see too that people do not *blindly* put things out there – Joe may have ‘a whole archive of stuff’ online but it is not stuff that if lost in some way would ‘ultimately affect his life’.

Putting personal data online is a *considered* act then, as can be seen in the sharing of family photos and videos, which our participants reported being the most ‘private’ category of personal data.

Fieldworker: Who has access to the videos on Vimeo?

Kit: It’s just family, isn’t it?

Tim: Yeah, family.

Kit: They tend to be on holiday, it’s not like there’s anything - if the kids were, say, running around in the nude on the beach, then I wouldn’t like it.

Tim: Yeah.

Kit: But I don’t think there’s anything like that really.

Tim: No. You do a sort of risk assessment don’t you? You know, how uncomfortable would I feel about that? I would feel uncomfortable if over the long term we were shoving pictures up of our kids. You’re sort of relinquishing control that they ought to have over keeping that private if they want to. You know, their history online, public, and they can’t get away from it. It’s sort of incumbent on us to be responsible enough to say, you know, they should have that choice. You kind of owe it to them to be a bit more responsible, rather than shoving everything online. But at the same time I don’t have any problems with the odd video or photo here and there.

As Kit and Tim make visible, consideration of what to put online turns upon ‘assessing’ its potential *impact* not only on self, as elaborated above by Joe, but on others. In this particular case we can see that in assessing whether or not to put family photos or videos online, ‘privacy’ is invoked with respect to parental responsibility and the foreseeable need to allow children to exercise their autonomy. More generally, we found that our participants routinely carried out *impact assessments* with respect to themselves and others, and this was particularly pronounced with respect to personal content posted on social media.

In saying that people routinely carry out impact assessments when putting personal content online we are not suggesting that they administer a formal procedure as defined, for example, by data protection bodies (e.g. ICO 2014). Rather, the impact of putting personal data online is assessed through a wide variety of ‘members’ methods’ (Button et al. 2015), glossed by accounts such as ‘would it ultimately affect my life’, ‘how uncomfortable would you feel’, ‘it be could quite embarrassing’, etc. The methodical application of reasoned judgements such as these inhabited our participant’s accounts about posting personal content on social media, and were complemented by discrete impact management practices centred on the use of multiple social media channels.

Alice: I use Facebook and WhatsApp, BabyCentre - interestingly that’s they only thing that I do anonymously. I don’t do it under my own name, because originally we were having trouble conceiving. I was a having a whole conversation about fertility problems I didn’t really want to have under my own name, and it’s still not really something I want associated with my name in terms of work or anything. I don’t want these connected. I don’t need that to be the thing that people get when I’m going to a job interview or whatever. At work I have an account for the council and one for the police and I don’t want those two to get jumbled up either. So I have me at home, me at work, and me at work when I’m doing stuff with the police, and I try and keep them all separate. I try quite hard to keep these things separate.

Alice’s comments encapsulate common practice amongst our participants, which sees them using multiple social media channels, and not infrequently anonymous social media channels where sensitive data is concerned, to enable the *‘separation’* of different cohorts, thereby limiting the potential impact of posting personal data online on the self. At the same time, and reflexively, the use of multiple social media channels enables the relationship-based tailoring of personal content. As Paul puts it,

Paul: I’ve got Facebook and I’ve got Twitter. I have a network of friends on Facebook that includes my family, some colleagues, things like that. On Twitter, even though I use my proper name, I don’t follow anybody that I know personally. I quite explicitly avoid connections with especially work colleagues on Twitter. I don’t follow any of them and I don’t want that link to be made, because I want to be able to behave in a different way on Twitter.

Thus we find in case after case that social media channels are exploited as *relationship-relevant channels*, though we note that there is no stability in choice of channels (e.g., that Twitter is used for a certain kind of cohort and Facebook for another). It is not simply the case that different channels are used for different purposes either, but that different channels are *tied* to different cohorts and the particular kinds of relationship that hold between their members.

The methodical ‘separation’ and ‘tying’ of cohorts to specific channels to manage the potential impact of posting personal content online also involves actively managing ‘follower’ relationships to maintain cohort separation and turns upon taken for granted expectations of data sharing. Thus, and for example, participants may use Facebook to post ‘public facing’ content to a broad cohort of followers, and use WhatsApp to post much more ‘personal’ content for a select few. In such circumstances data is shared on the basis of an assumed *right of disclosure*, which is taken to be commonly understood by recipients and further limits the potential impact of posting personal data online. Not that this always works.

Michel: One of the reasons why Carrie is not so sensitive about posting family photos on Facebook is because pretty well the only network who get to see that are family and friends. Whereas with me, the network who can actually see that includes work colleagues, some of whom I don’t even know very well even. I mean, we’ve had photos of me in fancy dress for instance on Facebook and it’s become clear that other people have had access to those things!

Fieldworker: So it’s other people’s stuff that you’re in and they’ve put up?

Michel: It’s never stuff that I share myself, no, ‘cause I don’t do that kind of stuff.

Carrie: I do, of fancy dress (laughs). Have you seen that one (Carrie holds up her iPad to Paul, and then turns it to show the fieldworker).

Fieldworker: (Laughs at photo of them both in fancy dress).

Michel: (Laughs).

Carrie: It’s stuff like that he doesn’t want me to put on.

Michel: This is the problem for me. I can control it all I like myself, but I have no control over what other people do.

It is tempting to see in Michel’s lack of control a violation of privacy at work but what actually concerns our participants in cases like these, and drives the separation of cohorts, is the *accountability* of their actions.

Sylvie: I tried for a while having people graded by their friendship status. So I’d have like real true friends, and then I had my work friends, who would ask me to be their friend but I felt kind of like socially awkward saying no to on Facebook, so I had them as acquaintances. It got really confusing. You know, someone might graduate from being an acquaintance to an actual friend but they still work with you, and then they come into work and say “oh I saw that picture of you at the park, it was really cute” and everyone else goes “what picture? I didn’t see that on Facebook.” So, I’ve given up on that. It just got really hard.

Whether occasioned by someone posting something personal about you online or you posting it yourself, it is not privacy per se that concerns people, but that they can be and occasionally are called to account for their actions by persons to whom they would rather not be accountable. Thus, and for example, Michel has in the past been called to account for wearing fancy dress by people he ‘doesn’t know very well’, just as Sylvie’s selective disclosure of photographs with Facebook friends led to her relationship with colleagues being called into account. In either case, and many more in our study, it was the *inappropriateness* of having to account for things said and done to people for whom they are of no business, with concomitant ‘uncomfortable’ and even ‘embarrassing’ affects, that concerned them. For many of the younger participants in our study the management of accountability through the management of follower relationships was especially bound up with concerns about not having to account for their online activity to the people they knew the best in everyday life, such as friends and family.

Fieldworker: And what kinds of people are following you [on Tumblr]?

Evelyn: Erm, very few people who know me in real life. Very, very few. There’s literally only Lionel and Tom [her brothers] I think who actually know me in real life. That’s probably why my - I’m more comfortable being more personal there ‘cause there’s less people I actually *know* personally.

Fieldworker: You’re sharing with people you don’t mind *see*ing that information obviously. If you got requests to follow you on Tumblr from people that you know in the real world outside of Lionel and Tom, how would you feel about that?

Evelyn: I would feel more uncomfortable certainly.

Cohort separation and channel tying is driven by the need to limit the impact of the digital on the accountability of persons and their actions, whether it is done to limit one’s own accountability (e.g., Michel’s or Sylvie’s or Evelyn’s) or others (e.g., Kit and Tim’s children’s). We thus find that our participants actively constrain the availability of personal data, not only in terms of restricting its distribution through the ad hoc selection of relationship-relevant channels, but also in terms of the *temporal durability* of data and/or the ability to delete personal content, which may drive the selection and use of specific channels for specific cohorts (both Snapchat and Twitter were frequently cited in respect of these issues). Where and when the need for ‘privacy’ does enter the equation then we find that our participants manage it one of two fundamental ways. Firstly by ‘channel switching’ and moving from written to oral media in particular (e.g., switching from Twitter to Skype), and secondly by simply *not* putting such materials online in the first place.

Sarah: Obviously I’m pregnant at the moment, but otherwise I had this [Fitbit app] to try and lose some weight and I didn’t really want people knowing, you know, to judge somehow how many more calories I was burning than them because I was so much more heavier than they are. I’d rather keep that myself really. Some people link it with their Facebook but my ideal nightmare would for that to be on Facebook saying, oh Sarah did this today or she’s lost two pounds or whatever. That for me is very separate information that don’t want really want to share with people. So I limit that.

Carrie: If I’m not happy to share it then it doesn’t go anywhere.

Michel: We share stuff about health in terms of weight and steps and things like that. We talk to each another, you know. Verbal communication suffices for sharing that kind of data, positive and negative.

Alice: I wouldn’t put anything on that I wasn’t happy for anybody to see. Managing real private stuff is – stuff shouldn’t exist, that’s the level of it. It doesn’t get written down. It doesn’t get put in a photo. It doesn’t exist. Definitely *do not put online*.

Just as we find that our participants have devised and use fine-grained methods for handling gateway control, thereby minimising the potential ‘attack surface’ for malicious or unintentionally damaging interaction in *their* networked world, so we also find that they have devised and use fine-grained methods for minimising the potential ‘attack surface’ on *their* personal data. They do not, then, put personal content online blindly, whether it be for purposes of managing collections of personal data or for purposes of sharing personal material with others, but through considered judgements where they assess the potential impact this might have on self and others. The methodical management of potential impact is particularly pronounced with respect to the distribution of personal content via social media. Here we find that our participants routinely employ social media channels as relationship-relevant channels to effect separations between the different cohorts they engage with. In tying different cohorts to different channels our participants effectively put people and personal content in different ‘buckets’ or ‘silos’, thereby limiting the potential ‘attack surface’ on their personal content, and with it *themselves* and implicated *others*. Again, it is notable that these methods are not wholly devised to manage ‘attacks’ on privacy. Indeed, ‘privacy’ was only occasionally invoked to account for the use of these methods. When ‘privacy’ was used it frequently glossed the primordial concern our participants have with the accountability of persons and their actions and the concomitant imperative to avoid the unpleasant effects of inappropriate disclosure. So again when we look at domestic privacy practices we find it glosses an array of *relationship management practices*, and this is also evident in our participant’s mundane interactions with the online world at large.

### Online interaction and privacy

Our participants were keenly aware that their interactions with the online world at large had personal consequences, particularly an increasing amount of targeted advertising based on their Internet activities (browsing, shopping, downloading, etc.). Many experienced the increasing flow of adverts resulting from their online interactions as a ‘bombardment’ and ‘nuisance’, but for others it was occasionally more personal than that:

Pat: It’s just a nuisance. It’s yet another window that’s in your face.

Sara: There’s one thing that worried me though. Do you remember that time – my family’s Jewish, and my uncle sometimes posts things, just once or twice, about searching for family in the Ukraine and stuff, and I was starting to find a shop selling everything Jewish coming up advertising on my page. So they’ve obviously made a connection that somewhere in the family there is somebody Jewish, and they’ve advertised that to me so that means obviously that it’s visible to somebody. It makes you very aware that people are watching what you’re doing. It’s like I was explaining to Hannah (teenage daughter) the other day. She was getting ads for pregnancy tests and she says, why am I getting this stuff. I said it’s targeted because you’re a teenage girl. And she said, but I’ve never gone on any site like that, I’ve never looked at anything. I said it doesn’t matter, they can tell by the type of sites that you do go on to – they can put you within an age group and sex group and so you’re targeted. She really doesn’t understand that even so. She says I go on gaming sites, I could be a boy. Yeah, you could, but even so the indications that you give are a flag to somebody.

Whether motivated by sheer ‘irritation’ or deeper concerns, such as the potential discriminatory consequences of being ‘tagged’ as Jewish, our participants adopted a variety of methods for managing interaction with the online world at large.

Thus we found that people routinely employed ‘throwaway’ email addresses (some in their own names, some not) to control the bombardment, the use of which turns on discerning the potential impact of handing over contact details when signing up to online services.

Joe: I’ve got a Gmail account, which I’ll occasionally give out to something that I know might generate spam or something.

Sarah: If I feel like it’s one of those where it’s like constantly gonna be, oh remember this or have you seen our latest sale or whatever, I’ll pick this old old email address that I don’t use for much else.

Lennie: I’ve got an old one that I don’t use for anything apart from signing up for things I know it’s going to tell somebody something. If it’s a service I know that at some point is going to sell that data onto somebody else, that’s the address it’s going to get.

Then, of course, we found the widespread use of ad blockers. Again, this was largely motivated by ‘irritation’ and ‘annoyance’ and the ‘convenience’ ad blockers provide in terms of smoothing out the ‘disruption’ to online interaction caused by a constant stream of ‘pop-ups’. However, some of the participants were also motivated by the ‘intrusive’ nature of online advertising:

Joe: If I browse something on Google - you know, when you’re properly logged in on the browser - then I’ll find if I’m looking at Facebook on my phone that these targeted adverts pop up, which are related to what I was browsing earlier on Amazon or Google or somewhere like that, and they’re appearing in the Facebook feed.

Fieldworker: Does that feel like an intrusion?

Joe: Yeah, it does a bit.

Participants reported using ‘whitelists’ to manage intrusion, with one (our IT support engineer) even implementing these at router level. In addition to throwaway emails, ad blockers and whitelists, we found that our participants were also managing the impact of the online world at large on everyday life by turning to privacy-preserving search engines, such as Startpage and Duck Duck Go, to reduce the number of ads they were being bombarded with.

We found too that our participants attempted to control the flow of personal data in the online world at large by ‘ticking’ or ‘unticking’ checkboxes to constrain the sharing of personal details, and by managing cookies. This included judging when to accept cookies and what the consequences of doing so might amount to.

Christine: I don’t always accept cookies. I accept cookies if I know I’m really wanting to get into this thing, but if I’m just skimming through something and they ask me that, then forget it.

Brian: I guess you if you want actually to go and buy …

Christine: Yeah, yeah.

Brian: Then you accept, or I accept, cookies.

Christine: Yeah.

Brian: As soon as you accept cookies obviously then they have your, you know, your patterns.

Cookies were not accepted blindly then, but turned upon the relevance of doing so to our participant’s activities. Thus, ‘just skimming’ through things for example did not warrant accepting cookies, whereas ‘buying something’ did, with the concomitant knowledge that in doing so one was making one’s ‘patterns’ of behaviour visible to third parties.

This visibility of behaviour patterns was not simply accepted as a ‘cost’ of being an inhabitant of the online world at large, however. Instead we found the widespread use of private browsing modes and the routine ‘dumping’ of caches by our participants to manage the uncertainties inherent in third parties having access to personal information.

Lewis: The browsers are configured to dump the cache when you close them. Wherever I can disable tracking I will do.

Fieldworker: So what motivates that then?

Lewis: It’s not knowing how third parties manage that information. If I don’t leave it on no one else can find it. So I took the decision to prevent them from being able to find it by removing it. So whenever you close the browser it will wipe your history and cache, and if you’ve not closed the browser properly or if the machine’s been hibernated rather than shut down I’ll go in once a week and clear the cache manually.

Clearing browser caches was commonplace amongst many of our participants, whether it was done through the use of plugins on a daily basis or manually on the basis of various contingencies (e.g., using credit cards online or doing routine digital housekeeping). In either case, clearing caches provided our participants with a means of reducing the visibility of online behaviour and thereby managing the potential impact of third party intrusions on everyday life.

It is also notable that the clearing of caches was also done to manage the visibility of online behaviour to those much closer to home.

Fieldworker: So do you clear caches, cookies or search histories?

Kit: The only time I’ve done it is when it’s like Tim’s birthday and I try to do things secretly so he doesn’t know. I put private browsing on and I – I’ve asked him before and he told me how to empty things.

Tim: Clear the cache, yeah. Yeah the only other times I could see mild embarrassment is if you’ve gone out and I’ve got Netflix to myself and then I’ll be like, right, good car chase film – when do I ever get to watch good car chase films? But then obviously it comes up, doesn’t it, you know, like next time you go on Netflix, you’ve been watching …

Kit: Oh! Hmmm.

Tim: So you can log onto Netflix and delete these things.

Fieldworker: And do you?

Tim: No I don’t actually. Well, if I did, I wouldn’t tell you, but I don’t. But I definitely wouldn’t answer that honestly if I did.

So, whether occasioned by someone’s birthday, or watching car chase films, or a host of other prosaic matters, caches were also routinely cleared to render online behaviour invisible, and thus unaccountable, to others within the home.

Again we can see that our participants employ fine-grained methods for reducing the potential ‘attack surface’, this time of the online world at large on everyday life. These methods exploit a range of technological mechanisms (throwaway email accounts, ad blockers, whitelists, privacy-preserving browsers, cookies, consent forms, cache clearing, and private browsing) and are employed to reduce ‘irritating’, ‘annoying’, ‘disruptive’, and occasionally disturbing ‘intrusions’. The methodical use of these mechanisms sees our participants working to manage the flow of personal data and the visibility of online behaviour in order to constrain what third parties can see and thus come to know about participant’s online behaviour, which in turn minimises unwarranted intrusions. We can also see that some of these mechanisms, particularly private browsing and cache clearing, are methodically employed to render online interaction invisible to others within the home. Once again, then, we find that these methods are not about privacy per se, but about accountability. Thus we find that our participants occasionally mask their online actions to reduce if not prevent those actions being called into account by those they live with, and do so for a host of accountable reasons implicated in the day-to-day conduct of interpersonal and indeed *intimate relationships* (surprising others, indulging in personal pleasures, etc.). We find too that a concern with accountability and relationship management also underpins our participant’s efforts to avoid constant ‘bombardment’ by the online world at large. In short, the online world at large is not one that our participant’s want to have an accountable relationship with, other than as an occasioned matter, e.g., when buying goods. However, even then they seek to constrain what can be seen and known about their online actions. Thus they work, and work methodically, to reduce the potential attack surface of the online world at large on their everyday lives.

## Privacy repacked

It might be said that our findings are unsurprising, obvious even, and merely detail what anyone knows about privacy in the networked world. That passwords are selectively shared, for example, or that particular social media channels are used to talk to particular people, or that private browsing is employed to mask online activities, is hardly news. However, reading our study under the auspices of common-sense would be to ignore what’s done in the doing and thus miss a grossly observable phenomenon that, in accounting for *their* digital privacy practices, our study participants’ have allowed *us* to make ‘instructably observable’.

“Ethnomethodology’s fundamental phenomenon and it’s standing technical preoccupation in its studies is to find, collect, specify, and make instructably observable the local endogenous production and natural accountability of … familiar society’s most ordinary organisational things … and to provide for them both and simultaneously, as objects, and procedurally, as alternate methods.” (Garfinkel 2002)

The organisational ‘object’ that our participants have allowed us to make instructably observable across twenty different households in two different countries is the concerted management of the potential ‘attack surface’ *of* the digital *on* everyday life occasioned by mundane interaction in and with the networked world. The methods providing for the local endogenous production of this organisational object are not, as previously noted, formal methods but an alternate array of members’ methods for managing the potential digital ‘attack surface’. It is worth noting for clarity’s sake that the notion of ‘attack surface’ with respect to the digital is usually invoked with respect to security, which is often treated as a panacea to privacy concerns, and the management of potential unauthorised access to hardware, software, firmware, and networks (Rosenquist 2015). Technologically the ‘attack surface’ is understood as the sum of the different points in a computational environment where an unauthorized user or ‘attacker’ could get into the environment, and could get data out (OWASP 2015). Members’ preoccupation with the digital ‘attack surface’ is rather different however, and focuses on managing cohort-dependent risks in the networked environment they inhabit, managing accountability in the sharing of personal data, and minimising intrusions into everyday life from the online world at large.

These preoccupations are articulated through a battery of fine-grained methods for managing multiple dimensions of the potential digital ‘attack surface’. Thus we find that members methodically employ passwords to manage the potential risks that attach to particular cohorts, e.g., to manage the risks of exposing ‘children’ to inappropriate content or of exposing ‘guests’ to embarrassing content or of revealing sensitive personal data to ‘tradesmen’. We see too, as a feature of the methodical use of passwords to manage the potential attack surface within the networked environments they inhabit, that members adopt and employ risk management strategies. Thus, the front door is a prima facie gateway. Then an array of gateways to devices, applications, and content come into operation on the basis of the accountable relationship persons have to household members, and which otherwise hold between household members themselves. Thus, members methodically employ cohort-relevant access controls to selectively control gateway access and thereby manage foreseeable cohort-dependent risks. We find that putting personal content online is, methodically, a considered act that turns upon assessing the potential impact of doing so, and that this is particularly pronounced with respect to personal content posted on social media. Here we find that members methodically employ multiple social media channels to enable the separation of the different cohorts they engage with. Thus different social media channels are methodically tied to different cohorts and the particular kinds of relationship that hold between their members. These are then methodically exploited as relationship-relevant channels to enable the relationship-based tailoring of personal content. We find too that cohort separation and channel tying is driven by the need to limit the impact of the digital on the accountability of persons and their actions, whether it is done to limit one’s own accountability or others. And we find that members methodically employ a range of technological mechanisms to control the flow of personal data within the broader networked world to reduce its ‘irritating’, ‘annoying’, ‘disruptive’, and occasionally disturbing ‘intrusion’ into everyday life, and to manage the visibility and subsequent accountability of their online actions both within the networked world and closer to home.

In fine-grained methodical detail it can be seen that members’ preoccupation with multiple dimensions of the potential digital ‘attack surface’ is not a preoccupation with the sum of the different points in a computational environment where an unauthorised user or ‘attacker’ could get into the environment, and could get data out. Rather, in managing the potential digital attack surface it is ‘attacks’ on the manifold *human relationships* members have and are implicated in that concern them in the interactions with the networked world, whether it be their relationships with their children, guests, friends of the kids, partners, their own friends, colleagues, followers, advertisers, retailers, etc. From the perspective of everyday life in the home, in which networked technology is embedded and used, the ‘attack surface’ looks very different to its technological construction. The abiding concern is not with hardware, software, firmware or networks, but *people* and the impact the networked world might have on their interpersonal relationships. Our study makes it instructably observable that the concerted management of potential attacks on these relationships is a major preoccupation in members’ interaction with the networked world. Thus they have devised and employ their own methods for organising interaction in the networked world that reflect this preoccupation. This is not to say that all people used the same array of methods - the organisational object our study identifies is *locally* produced – but that the inhabitants of the networked world exploit elements of a distinctive methodology, consisting of discrete methods and logics governing their use, according to individual circumstance. We suspect it is an evolving methodology, one that will grow more sophisticated and even adapt as the networked world grows in complexity. Insofar as its elements are employed by the study cohort we take it that it applies more generally too (Crabtree et al. 2013), and is thus something others can go and see for themselves and further elaborate in future studies.

When we examine the methods that members have devised to manage the potential attack surface of the digital on their interpersonal relationships it can be seen too that members are *not* primarily concerned with attacks on their privacy. The emphasis we have placed on such matters as risk management, assessing impact, and managing the visibility of action online may invite the reader to conclude that our study speaks directly to the notion of ‘privacy calculus’ but, as our study makes perspicuous, there is a great deal more to the management of risk in the networked world than calculating privacy trade-offs: child safety, being a good host, doing things for others, arranging secret surprises, etc. Rarely are such matters concerned with privacy, but rather with the methodical management of interpersonal relationships in which the networked world is implicated. Similarly, it might be concluded that in emphasising relationship management our study speaks to key concepts of privacy: to boundary management and norms controlling the appropriate flow of personal data within human relationships. We concede it may be possible to read our study that way. However, and to borrow from Taylor’s reflection on the hazards of using a priori theories and concepts in HCI, we are not convinced that this is the case:

“I’ve suggested this can lead to confusion over the substance and purpose of the work: whether it succeeds in describing what, exactly, is going on in a setting or whether it is, instead, engaging in some protracted disciplinary dialogue. Often the latter is confusingly presented as the former … I’ve argued that HCI runs the risk of repeating this same confusion. … and is thus in danger of losing sight of or worse still misrepresenting the phenomena it seeks to report on and design for.” (Taylor 2011)

In saying this it is not Taylor’s aim to “doggedly dismiss” a priori theory or question the value of importing concepts from other disciplines, but rather to point out that such devices “really tell us very little about what is going on ‘out there’.” Hence our reservation about reading our studies in light of prevailing concepts of privacy. Indeed, in trying to get to grips with the phenomenon our study revealed to us, we found that concepts and theories of privacy were akin to the foliage that stops us seeing ‘what is going on’ and thus hides the animal beyond. The animal beyond in this case is members’ preoccupation with human relationships in the networked world and the abiding need to manage potential ‘attacks’ on their accountable conduct. ‘Privacy’ obscures this methodological preoccupation, with the attendant risk of engaging us in a protracted disciplinary dialogue that loses sight of and misrepresents what, exactly, is going on ‘out there’.

So what of privacy? Are we saying it should be dispensed with? That it does not matter? No. But we are suggesting, as others before us have suggested, that “privacy is more of a concern for us as researchers than it is of practical concern to families” (Brown et al. 2007) and, indeed, households more generally. We find too in attempting to explore people’s digital privacy practices, as Barkhuus (2012) puts it, that privacy is “more complex than just *concern*” where this is understood to be about measuring various factors that impact the disclosure of personal data. It is not simply that privacy concerns “are not easily measurable”, as Barkhuus also points out, but rather that when the concept of privacy is invoked to elicit data from people it “introduces the complexity of multiple interpretations of the word” (ibid.). When we look to see what those multiple interpretations consist of it becomes apparent that, like the concept of ‘work’, ‘privacy’ elaborates a *polymorphous* array of mundane activities implicated in the conduct of manifold human relationships (Schmidt 2011). So just as Schmidt points out, in drawing on the work of philosopher Gilbert Ryle (1971) to elaborate the concept of work, that “there is nothing which must be going on in one piece of work which need be going on in another” and that “nothing [therefore] answers to the general description ‘what work consists of’”, we find the same applies to privacy. The upshot of this is that ‘privacy’ *dissolves* into a heterogeneous array of mundane practices and local concerns that are not primarily to do with the disclosure of personal data, but with managing ***who*** gets to access what devices, applications, and content. Now this might sound like a privacy concern on the face of it, but to construe it this way would be to wilfully ignore and gloss over a whole raft of mundane practical matters to do with the day-to-day management of human relationships: matters to do with sharing a home with others, being in family, being a responsible parent, a good host or neighbour, managing collections of household data, making sure the kids don’t destroy content or get exposed to content that might upset them, engaging with the wider network of family and friends, etc. These mundane concerns, and a great many more, were surfaced when we attempted explore members’ digital privacy practices and in surfacing them what we found at work was not a concern with privacy per se, but a concern to manage the potential ‘attack surface’ of the digital on the manifold relationships implicated in their everyday lives.

Members’ methodological preoccupation with the digital ‘attack surface’ focuses, as noted above, on managing cohort-dependent risks in the networked environment they inhabit, managing accountability in the sharing of personal data, and minimising intrusions into everyday life from the online world at large. We have seen how a variety of technological mechanisms have been methodically appropriated by members to manage their relationship with the networked world beyond their door. While design efforts continue to better enable this through the design of vendor relationship management tools (e.g., McKay 2010), personal data stores (e.g., de Montjoye et al. 2012) and personal networked services (e.g., Chaudry et al. 2015), our study suggests there is a need to support the management of interpersonal relationships too: relationships within households and between household members, for example, as well broader interpersonal networks that extend beyond the confines of the home. Clearly members have devised methods for managing these relationships in the networked world as it stands. However, as it evolves and new technologies are built into it there is need to consider how design might support the management of cohort-dependent risks and accountability and assist people in minimising, if not preventing, unwanted intrusions into everyday life from the online world at large. This becomes particularly prescient with respect to the anticipated impact of the Internet of Things on everyday life, where managing ***who*** gets to access what devices, applications, and content is foreseeably a matter of widespread concern. There is a need, then, for design to complement or extend its interest in building privacy mechanisms with an explicit focus on the development of mechanisms that enable members to manage the potential attack surface of the digital on their day-to-day relationships in a massively networked world. Simply put, the focus needs to move from the individual to the social, and where content is concerned, from controlling the flow of data to managing the human relationships involved in making the data flow.

### Limitations of the study (?)

There is one final methodological issue that needs to be addressed. A concern was raised that the people we spoke to were largely secure and, unlike many vulnerable persons, not unduly fearful that various aspects of their lives might be exposed. It seems to us, however, that far from highlighting the limitations of our study, the ‘fear’ argument rather *underscores* it. Everyone – be it an illegal alien, victim of domestic violence, a wife who simply wants to plan a surprise, a person who wishes to avoid being called to account for their non-work activities, a couple who enjoy pornography together, or a Jewish family exposed to ads surfacing their religion – has reason to fear exposure. Fear of exposure is not the sole preserve of the vulnerable, but a mundane matter driven by a heterogeneous array of local relational concerns, which is to say that ‘fear’ is specific and occasioned with respect to *just what* might be exposed and to *just who:* an illegal alien’s presence in the country to the authorities, a battered woman’s location to a violent partner, a wife’s birthday surprise to her husband, a person’s non-work activities to work colleagues, a couple’s pornography stash to guests, a family’s Jewish heritage to Facebook, etc. Contrary to the view that our study might be limited because our sample is comfortable and ignores those at the margins, we take it that it surfaces a generic phenomenon: that members have an abiding methodological concern with managing the potential attack surface of the digital on their everyday lives. That is an empirical proposition, and the ‘fear’ argument neither limits nor negates it. Rather, it begs the question, should one wish to pursue it, as to how those at the margins of society exercise that methodological concern locally, in the course of conducting their everyday lives, just as others do?

## Conclusion

Privacy is widely seen and treated as the ‘silver bullet’ to the global ‘crisis in trust’ that currently affects the digital ecosystem. Its preservation is seen as a key means of rebalancing the asymmetry of power that favours public and private institutions over individuals and thereby creates barriers to economic development. Within CSCW, and the related areas of HCI and Ubiquitous Computing, this concern with privacy leads to a pronounced interest in providing ‘users’ with enhanced control mechanisms to manage the flow of personal data in the digital economy.

Our study of digital privacy practices in the home suggests that within a domestic context at least ‘privacy’ glosses members’ methodological preoccupation with the management of the potential ‘attack surface’ *of* the digital *on* everyday life. This concern with the potential digital attack surface is not with hardware, software, firmware or networks, and the sum of the different points in a computational environment where an unauthorised user or ‘attacker’ could get into the environment, and could get data out. Rather it is a concern with the day-to-day management of cohort-dependent risks in the particular networked environments members inhabit, with managing the accountability of their own and others’ actions in the sharing of personal data, and with managing intrusions into everyday life from the online world at large. It is a concern with *people* and the impact the networked world might have on their interpersonal affairs, and thus a concern with managing ***who*** gets to access what devices, applications, and content in the face of a whole raft of mundane practical matters to do with the day-to-day management of *human relationships*.

Seen from the perspective of everyday life, privacy thus *dissolves* into a heterogeneous array of mundane activities, practices and concerns to do with such things as sharing a home with others, being in family, being a responsible parent, etc. It is managing the potential ‘attack surface’ of the digital on these matters that concerns members, and leads us to the conclusion that privacy is not a silver bullet. Resolving the global crisis in trust will turn as much upon providing adequate relationship management mechanisms that enable people to handle cohort-dependent risk, accountability and intrusion in a massively networked world, as it will turn upon individual privacy mechanisms. As others before us have suggested, “relationships are in essence defined by what type of information we share with one another” (Barkhuus 2012). We would add to that they are also defined by who we allow our devices, applications, and content to be accessed by and the mundane relational concerns that drive that. Enabling people to manage their relationships within the home and with others beyond it is, we suggest, critical to building trust in and fostering engagement with the emerging digital ecosystem.

## Footnotes

1 In review it was suggested that our review of the literature elaborates the state of the art in the area but falls short of providing a critical perspective on the subject of privacy. We agree. The literature review is, as noted, cursory and restricted to identifying discrete trends. Saying more than this at this point would displace the argument set out below on the basis of the empirical study that follows. However, we note that in respect of this study the reviewer who raised this issue says, “in suggesting repacking ‘privacy’ as matter of ‘supporting people’s evident interest in managing their relationships in and with the networked world’ as opposed to its traditional framing as a simple matter of trade-offs, this paper offers some new ways of designing for ‘privacy’ that people are faced with when using shared virtual and physical spaces in a networked reality.”
